# Relationship between cardiorespiratory phase coherence during hypoxia and genetic polymorphism in humans

**DOI:** 10.1113/JP278829

**Published:** 2020-02-26

**Authors:** Gemma Lancaster, Tadej Debevec, Gregoire P. Millet, Mathias Poussel, Sarah J. Willis, Minca Mramor, Katja Goričar, Damjan Osredkar, Vita Dolžan, Aneta Stefanovska

**Affiliations:** ^1^ Department of Physics Lancaster University Lancaster UK; ^2^ Faculty of Sport University of Ljubljana Ljubljana Slovenia; ^3^ Department of Automation Biocybernetics and Robotics Jožef Stefan Institute Ljubljana Slovenia; ^4^ Institute of Sport Sciences University of Lausanne Lausanne Switzerland; ^5^ Department of Pulmonary Function Testing and Exercise Physiology CHRU de Nancy Nancy France; ^6^ University Children's Hospital University Medical Center Ljubljana Ljubljana Slovenia; ^7^ Pharmacogenetics Laboratory Institute of Biochemistry Faculty of Medicine University of Ljubljana Ljubljana Slovenia

**Keywords:** cardiovascular dynamics, *CAT*, heart rate variability, hypoxia, *NOTCH4*, periodic breathing, wavelet analysis

## Abstract

**Key points:**

- High altitude‐induced hypoxia in humans evokes a pattern of breathing known as periodic breathing (PB), in which the regular oscillations corresponding to rhythmic expiration and inspiration are modulated by slow periodic oscillations.
- The phase coherence between instantaneous heart rate and respiration is shown to increase significantly at the frequency of periodic breathing during acute and sustained normobaric and hypobaric hypoxia.
- It is also shown that polymorphism in specific genes, *NOTCH4* and *CAT*, is significantly correlated with this coherence, and thus with the incidence of PB.
- Differences in phase shifts between blood flow signals and respiratory and PB oscillations clearly demonstrate contrasting origins of the mechanisms underlying normal respiration and PB.
- These novel findings provide a better understanding of both the genetic and the physiological mechanisms responsible for respiratory control during hypoxia at altitude, by linking genetic factors with cardiovascular dynamics, as evaluated by phase coherence.

**Abstract:**

Periodic breathing (PB) occurs in most humans at high altitudes and is characterised by low‐frequency periodic alternation between hyperventilation and apnoea. In hypoxia‐induced PB the dynamics and coherence between heart rate and respiration and their relationship to underlying genetic factors is still poorly understood. The aim of this study was to investigate, through novel usage of time–frequency analysis methods, the dynamics of hypoxia‐induced PB in healthy individuals genotyped for a selection of antioxidative and neurodevelopmental genes. Breathing, ECG and microvascular blood flow were simultaneously monitored for 30 min in 22 healthy males. The same measurements were repeated under normoxic and hypoxic (normobaric (NH) and hypobaric (HH)) conditions, at real and simulated altitudes of up to 3800 m. Wavelet phase coherence and phase difference around the frequency of breathing (approximately 0.3 Hz) and around the frequency of PB (approximately 0.06 Hz) were evaluated. Subjects were genotyped for common functional polymorphisms in antioxidative and neurodevelopmental genes. During hypoxia, PB resulted in increased cardiorespiratory coherence at the PB frequency. This coherence was significantly higher in subjects with *NOTCH4* polymorphism, and significantly lower in those with *CAT* polymorphism (HH only). Study of the phase shifts clearly indicates that the physiological mechanism of PB is different from that of the normal respiratory cycle. The results illustrate the power of time‐evolving oscillatory analysis content in obtaining important insight into high altitude physiology. In particular, it provides further evidence for a genetic predisposition to PB and may partly explain the heterogeneity in the hypoxic response.

## Key points

- High altitude‐induced hypoxia in humans evokes a pattern of breathing known as periodic breathing (PB), in which the regular oscillations corresponding to rhythmic expiration and inspiration are modulated by slow periodic oscillations.
- The phase coherence between instantaneous heart rate and respiration is shown to increase significantly at the frequency of periodic breathing during acute and sustained normobaric and hypobaric hypoxia.
- It is also shown that polymorphism in specific genes, *NOTCH4* and *CAT*, is significantly correlated with this coherence, and thus with the incidence of PB.
- Differences in phase shifts between blood flow signals and respiratory and PB oscillations clearly demonstrate contrasting origins of the mechanisms underlying normal respiration and PB.
- These novel findings provide a better understanding of both the genetic and the physiological mechanisms responsible for respiratory control during hypoxia at altitude, by linking genetic factors with cardiovascular dynamics, as evaluated by phase coherence.

## Introduction

Respiration occurs spontaneously through the coordinated action of many finely balanced processes within the body. The respiratory system contains chemoreceptors that continuously monitor the levels of blood oxygen and carbon dioxide, which in turn influence the breathing rate and the volume of the lungs. Continuous feedback mechanisms usually maintain blood oxygen and carbon dioxide levels within narrow ranges, but in extreme environments such as high altitudes, or when affected by certain diseases, these mechanisms can overcompensate or fail. One consequence of this is an amplitude modulation of respiration known as periodic breathing (PB).

PB is a well‐known phenomenon that may arise in healthy individuals at high altitudes (Berssenbrugge *et al*. 1983). It is defined as repetitive oscillations in breathing amplitude, with each cycle containing a period of hyperventilation followed by a period of apnoea. PB has been widely reported during sleep at altitude, causing significant sleep disturbance (Ainslie *et al*. 2013), but has also been observed during the awake state, during both rest and exercise (Hermand *et al*. 2014; Garde *et al*. 2015). PB‐type respiration is not unique to those ascending to high altitudes, with similar breathing patterns being observed at sea‐level in sleep apnoea (Garde *et al*. 2015), newborn infants (Kelly *et al*. 1985) and pathological conditions such as heart failure. The breathing pattern observed in the latter is known as Cheyne–Stokes respiration (Cherniack & Longobardo, 1973).

PB develops due to changes in the complex interactions between peripheral and central chemoreflexes and cerebral blood flow (Ainslie *et al*. 2013) induced by environmental changes (Burgess *et al*. 2008). Reduced partial pressure of oxygen in the air at high altitudes may induce hypoxaemia, leading to hyperventilation in order to rapidly increase blood oxygen levels. Increased ventilatory sensitivity during hypoxia causes ‘overshooting’ of this feedback system, and thus over‐hyperventilation leads to hypocapnia. This then reduces the respiratory drive and leads to a period of apnoea. Apnoea will then cause blood CO_2_ levels to rise, and the cycle repeats, leading to periodic breathing (Hernandez & Patil, 2016). The extent of PB increases with the duration and severity of hypoxia (Ainslie *et al*. 2013), and the cycle length decreases with increasing altitude (Küpper *et al*. 2008).

The altered respiratory pattern due to PB directly affects other systems within the body, including the heart rate and the blood flow. In normal conditions, respiration directly affects the heart rate, in a process known as respiratory sinus arrhythmia (RSA) (Hirsch & Bishop, 1981; Elstad *et al*. 2018). RSA usually manifests in the frequency spectrum at the respiration frequency, near 0.25 Hz. Lower‐frequency spectral components have also been observed in heart rate variability (HRV) during PB and are directly related to respiration (Leung *et al*. 2003); RSA is modulated by hypercapnia but not mild hypoxaemia (Tzeng *et al*. 2007). These interactions between breathing and heart rate have been used to quantify PB (Garde *et al*. 2015), thus providing the basis for an automatic method for characterising PB.

There is a great deal of variability in the response to hypoxia, including the onset of PB, whose origin is still poorly understood. The variation cannot be explained by environmental factors alone. There is also a genetic component (Strohl, 2003). In two different mouse strains exposed to the exact same conditions, only one exhibited post‐hypoxic PB. It is now accepted that genetics plays an important role in the hypoxic ventilatory response (HVR), demonstrated in humans by family and population studies (Weil, 2003; Teppema & Dahan, 2010; Hennis *et al*. 2015). Although genetics is closely linked to the hypoxic response, the exact genes involved and their specific contributions remain to be elucidated.

The aim of the present study was to examine the oscillatory characteristics of hypoxic periodic breathing, both in normobaric and hypobaric conditions, with particular focus on the phase coherences and phase shifts among oscillations in the heart rate, respiration and microvascular flow. Furthermore, we hypothesized that common functional genetic polymorphisms in selective antioxidative (*SOD2*, *CAT* and *GPX*) and neurodevelopmental (*NOTCH4* and BDNF) genes are statistically linked to features of these oscillations, such as their phase coherences. This could then elucidate whether genetic factors contribute to the heterogeneity of PB incidence among individuals during hypoxia.

## Methods

### Ethical approval

The study protocol was registered at ClinicalTrials.gov (NCT02780908) and approved by the National Committee for Medical Ethics of the Republic of Slovenia (0120‐101/2016‐2). In addition to this ethical approval, which covered the whole experiment (all laboratories in Slovenia and France), local approval was obtained through the Ifremmont (Institut de formation et de recherche en médecine de montagne) for the experiments conducted in Chamonix (France). All experimental procedures were conducted in line with the standards set by the latest version of the *Declaration of Helsinki*. The participants were given detailed verbal and written instructions regarding all experimental procedures and any possible risks involved prior to the experimental protocol. Written informed consent was obtained from all participants before the start of the first measurements.

### Experimental design

Twenty‐two healthy subjects were recruited for the study and monitored in five different environments: normobaric normoxia (NN), normobaric hypoxia (both acute (NH_a_) and after 6 h of exposure (NH)), and hypobaric hypoxia (both acute (HH_a_) and after 6 h exposure (HH)). All locations and measurement conditions are shown in Table 1.

**Table 1:** Recording location information for all groups

Group	Location	Sample size	Altitude (m)	$F_{{\mathrm{IO}}_{2}}$ (%)	$P_{{\mathrm{iO}}_{2}}$ (mmHg)	BP (mmHg)
NN	Ljubljana (normoxia)	22	300	0.21	146	743
NH_a_	Ljubljana (hypoxia)	22	∼3800	0.13	91	743
NH	Planica	18	∼3840	0.14	90	684
HH_a_	Aiguille du Midi	16	3842	0.21	90	478
HH	Aiguille du Midi	13	3842	0.21	90	478

In acute hypoxic states, recording commenced at the onset of hypoxia. Data from prolonged hypoxic states were recorded following 6 h of acclimatisation. BP, barometric (ambient) pressure; $F_{{\mathrm{IO}}_{2}}$, fraction of inspired O_2_; HH, prolonged hypobaric hypoxia (real/terrestrial altitude); HH_a_, acute hypobaric hypoxia (real/terrestrial altitude); NH, prolonged normobaric hypoxia (simulated altitude); NH_a_, acute normobaric hypoxia (simulated altitude); NN, controls (normobaric normoxia); $P_{{\mathrm{iO}}_{2}}$, partial pressure of inspired O_2_.

Participants were all male, aged 21 ± 1.6 years. BMI (22.5 ± 3.0 kg/m^2^), blood pressure (systolic (126.2 ± 6.6 mmHg), diastolic (77.8 ± 8.3 mmHg)), body fat (19.9 ± 7.7%), height (1.75 ± 0.08 m) and body mass (68.94 ± 7.48 kg) were also recorded during the first preliminary visit to Ljubljana, with all subjects being well‐rested. Participants were first tested in a random order during normobaric normoxia (NN) and acute normobaric hypoxia (NH_a_) in a laboratory in Ljubljana, situated at an altitude of 300 m. During the normoxic exposure the participants inspired ambient air, while during the acute normobaric hypoxic (NH_a_, simulated altitude) exposure they inspired a humidified hypoxic gas mixture ($F_{{\mathrm{IO}}_{2}}=0.13$, $P_{{\mathrm{iO}}_{2}}=91\;\mathrm{mmHg}$; Table 1) from a 200 litre Douglas bag via a two‐way low resistance valve (2700 NRBV; Hans Rudolph Inc., Shawnee, KS, USA) connected to an oro‐nasal mask (Vmask, Hans Rudolph). The prolonged normobaric hypoxic (NH) session was performed in the normobaric hypoxic facility in Planica (Rateče, Slovenia). The participants remained in the laboratory under normobaric hypoxic conditions ($F_{{\mathrm{IO}}_{2}}=0.14$, $P_{{\mathrm{iO}}_{2}}=90\;\mathrm{mmHg}$; Table 1) for 6 h prior to the measurements. As detailed elsewhere (Debevec *et al*. 2014), the normobaric hypoxic environment within the laboratory was generated and maintained using a Vacuum Pressure Swing Adsorption system (b‐Cat, Tiel, The Netherlands). Acute and prolonged exposure sessions in hypobaric hypoxia (HH, terrestrial altitude) were subsequently performed at a high‐altitude laboratory situated at the top of Aiguille du Midi Mountain (Chamonix, France; see Table 1 for details). The participants were taken to the laboratory using a cable car without any physical exercise and performed an acute test immediately upon arrival (HH_a_) and the prolonged exposure test after 6 h of altitude exposure (HH).

For safety purposes a medical doctor was present during all hypoxic exposures conducted in Ljubljana, Planica and Aiguille du Midi laboratories. The participants were constantly equipped with their personal finger oximetry device (3100 WristOx, Nonin Medical Inc., Plymouth, MN, USA) in order to monitor heart rate and capillary oxyhaemoglobin saturation responses. Should the participants experience any adverse physiological effects they would be disconnected from the hypoxic gas mixture (Ljubljana laboratory), removed from the hypoxic room (Planica laboratory) or would descend by the cable car to the lower altitude (Aiguille du Midi laboratory). During prolonged normobaric hypoxic exposures in Planica the participants also wore portable ambient O_2_ concentration analysers (PGM‐1100, RAE Systems, San Jose, CA, USA) which activated a safety audible alarm if the O_2_ levels decreased below the pre‐set levels.

Electrocardiograms (ECG), respiration and skin blood flow at both wrists were measured for 30 min for each subject in each condition. Respiration was measured using an adjustable Velcro belt incorporating a TSD201 conductance transducer (Biopac Systems Inc., Goleta, CA, USA). The recorded signal is proportional to the expanding and contracting volume of the thorax as generated by breathing. Blood flow signals were measured using laser Doppler flowmetry (LDF) (DRT4, Moor Instruments, Axminster, UK). Before data analysis, LDF signals were inspected, and movement artefacts were removed using linear interpolation. ECG and respiration signals were recorded at a sampling frequency of 1200 Hz, and blood flow signals at a sampling frequency of 40 Hz. Not all participants attended all measurements; sample sizes are shown in Table 1.

All datasets with simultaneously recorded signals for each participant and each condition (normoxic and hypoxic), including the genetic information collected from each participant (for details see below), are available at the Publications and Research (Pure) portal on Lancaster University's research information management system at https://doi.org/10.17635/lancaster/researchdata/333.

### Genetics

For each subject a 5 ml sample of peripheral blood was collected into a tube with sodium citrate and stored at −70°C. Genomic DNA was isolated from peripheral blood leukocytes using the FlexiGene DNA kit (Qiagen, Hilden, Germany) according to the manufacturer's instructions. *SOD2* rs4880, *CAT* rs1001179, *BDNF* rs6265 and *NOTCH* rs367398 polymorphisms were genotyped using KASPar assays (KBiosciences, Hoddesdon, UK and LGC Genomics, Hoddesdon, UK) while *GPX1* rs1050450 polymorphism was genotyped using TaqMan (Thermo Fisher Scientific, Waltham, MA, USA) according to the manufacturer's instructions.

### Time–frequency analysis

The inherent time variability of biological systems means that to understand the state of the system properly, the dynamics must be recorded over a sufficiently long interval to characterise the oscillations present. It is also necessary to apply analysis methods that, unlike the Fourier transform, take into account frequency variations over time.

Wavelet analysis is used to obtain time–frequency representations of data. It is an optimal choice for the analysis of biological oscillations, enabling a sound analysis when there are both high‐ and low‐frequency components present, due to the logarithmic frequency resolution and the adaptive window size. It has already been applied successfully in previous studies of periodic breathing in new‐born infants (Mohr *et al*. 2015), during sleep apnoea (Figliola & Serrano, 1997; Figliola *et al*. 2003) and during exercise (Garde *et al*. 2015).

**Figure 1:**
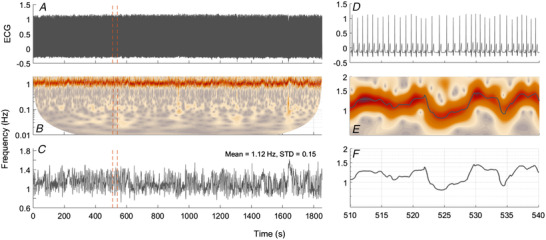
Example extraction of instantaneous heart rate from the ECG signal *A*, whole ECG time series. *B*, wavelet transform of the ECG signal in *A*. A clear oscillatory mode around the expected heart rate (1 Hz) can be seen. *C*, the time series of instantaneous frequency extracted from the wavelet transform using ridge extraction (for details see main text). *D*, 30 s portion of the ECG signal shown in *A*, marked by vertical orange dashed lines. *E* and *F*, the corresponding wavelet transform (*E*) and extracted heart rate (*F*). [Color figure can be viewed at wileyonlinelibrary.com]

The continuous wavelet transform (CWT) is,

$$g\;\left(s,t\right)=\;\frac{1}{s}\int_{-\infty }^{\infty }\psi \left(\frac{u-t}{s}\right)g\left(u\right)du,$$

where g(s,t) are complex coefficients obtained from the convolution of the wavelet ψ with the time series. ψ is the mother wavelet, which is scaled by the factor *s* and translated in time by *t*. Here, we use the complex Morlet wavelet. For each point in time, and for each frequency, we obtain both a wavelet amplitude and a wavelet phase. We define wavelet power as the square of the wavelet amplitude. Biological oscillations do not usually have perfectly constant frequency; rather, they vary in time around certain values. The frequency at each moment in time is called the *instantaneous frequency*. The wavelet amplitudes provide information about the presence of oscillations at specific frequencies in the data and describe how the instantaneous frequencies change over time. This information can be obtained by ridge extraction (Iatsenko *et al*. 2016), allowing the dynamics of individual oscillators to be isolated, and then used in further analysis. Figure 1 demonstrates the extraction of the time‐varying heart rate from the ECG signal. The frequency of the heartbeat is traced in time, and this time series then specifies the instantaneous heart rate (IHR).

**Figure 2:**
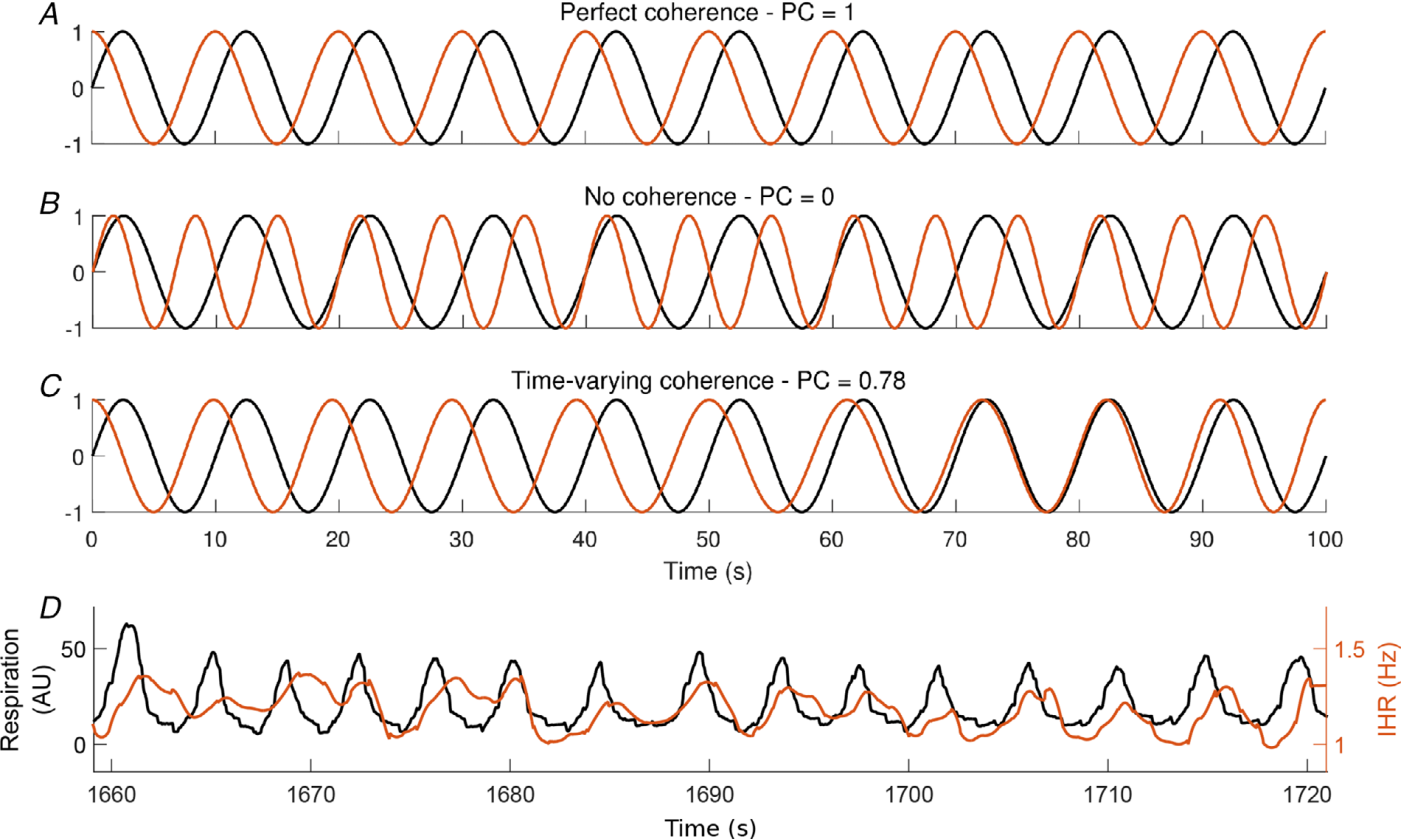
Examples of phase coherence values In *A–C*, the black curve shows a sine wave of constant frequency 0.1 Hz. *A*, the orange line shows a sine wave of the same frequency, 0.1 Hz, so the phase coherence (PC) at 0.1 Hz is equal to 1, since the phase difference is constant over all time. *B*, the orange wave is a sine wave of different frequency from that of the black wave, so the phase coherence at 0.1 Hz tends towards zero. *C*, the orange wave has time‐varying frequency centred around 0.1 Hz: at some times the orange wave is coherent with the black, but less so at other times, and so the phase coherence value at 0.1 Hz is less than 1 but more than 0. In reality, the frequency of a signal from a living system is not constant but varies over time. *D*, example of a real respiration signal (black) and the simultaneous instantaneous heart rate (IHR, red) signal after it has been extracted from the wavelet transform of the ECG as described in Fig. 1. It is clear that there are oscillations in both signals, and that they are oscillating at a similar frequency. [Color figure can be viewed at wileyonlinelibrary.com]

It is also possible to extract the phase associated with each frequency at each time, information that forms the basis of *phase coherence*. Given two signals, phase coherence evaluates how constant their phase difference at each frequency remains over time. If the phase difference is almost constant, the phase coherence will tend towards 1. A monotonically varying phase difference will result in a phase coherence closer to zero. An example calculation of the phase coherence $C_{\Phi }$ is shown in Fig. 2*A*–*C*, in which $C_{\Phi }$ is defined as (Bandrivskyy *et al*. 2004; Bernjak *et al*. 2012; Sheppard *et al*. 2012):

$$C_{\Phi }\left(f_{k}\right)=\sqrt{{〈\cos\Delta \Phi_{k,n}〉}^{2}+{〈\sin\Delta \Phi_{k,n}〉}^{2},}$$

Here $\Delta \Phi_{k,n}\;=\;\Phi 2_{,k,n}-\Phi 1{,}_{k,n}$ and $\Phi 1{,}_{k,n\;}$and $\Phi 2{,}_{k,n}$ are the instantaneous phases calculated at each time $t_{n}$ and frequency $f_{k}$.

We have investigated the relationship between breathing and heart rate, including the phenomenon known as respiratory sinus arrhythmia (RSA). An example of this phenomenon can be seen in Fig. 2*D*. There are clear oscillations of a similar frequency in both signals. Phase coherence quantifies the extent to which these two oscillations are related.

The IHR for each subject in each condition was extracted from the ECG signal using ridge extraction in the frequency range 0.5–2 Hz. The ECG signals were resampled to 40 Hz before the extraction. The mean and standard deviation of the IHR time series were calculated for each subject. The instantaneous respiration rate was extracted in a similar way, but in the frequency range 0.145–0.6 Hz.

Even in completely incoherent systems, non‐zero coherence values may still be obtained, especially at low frequencies where a reduced number of available cycles results in artificially increased coherence. To correct for this bias, and to ascertain whether obtained coherence values are significant, surrogate data testing is required (Theiler & Prichard, 1996; Schreiber & Schmitz, 2000; Sheppard *et al*. 2012; Lancaster *et al*. 2018). Surrogates attempt to mimic all the properties of the signal except the one being tested, in this case the possible phase relationships between the signals.

### Step‐by‐step algorithm

The functions used during the analysis are available to download as part of the Multiscale Oscillatory Dynamics Analysis (MODA) toolbox from https://github.com/luphysics/MODA.

#### Heart rates

**Figure 3:**
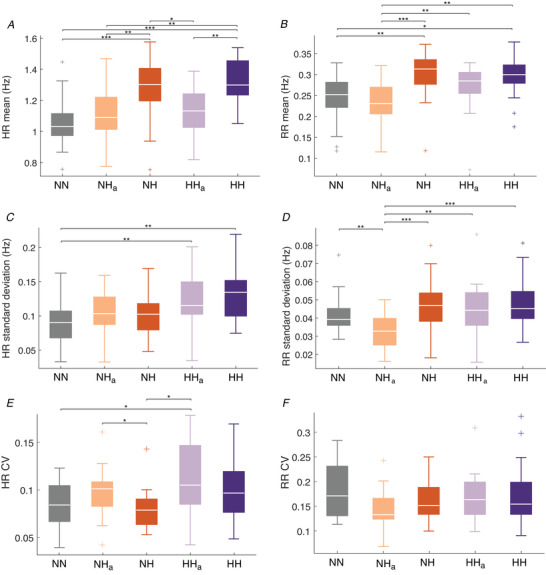
Boxplots depicting heart and respiration rates for all groups On each box, the central mark indicates the median, and the bottom and top edges of the box indicate the 25th and 75th percentiles, respectively. The whiskers extend to the most extreme data points not considered outliers, and the outliers are plotted individually using the ‘+’ symbol. *A*, mean heart rate (*P* = 0.0000). *B*, mean respiration rate (*P* = 0.0001). *C*, standard deviation of the heart rates (*P* = 0.0151). *D*, standard deviations of the respiration rate (*P* = 0.0002). *E*, coefficient of variation of heart rate (*P* = 0.0453). *F*, coefficient of variation of the respiration rate (*P* = 0.2931). *P*‐values are from the Kruskal–Wallis test. NN, control conditions (normobaric normoxia); *n* = 22; NH_a_, acute normobaric hypoxia (simulated altitude); *n* = 22; NH, normobaric hypoxia; *n* = 18; HH_a_, acute hypobaric hypoxia (real/terrestrial altitude); *n* = 16; HH, hypobaric hypoxia; *n* = 13. The same subjects were included on all 5 occasions, although some dropped out because they could not travel from Ljubljana, Slovenia either to Planica, Slovenia or to Aiguille du Midi, France, or felt unwell because of prolonged hypobaric hypoxia at Aiguille du Midi. ^*^ *P* < 0.05, ^**^ *P* < 0.01, ^***^ *P* < 0.001. [Color figure can be viewed at wileyonlinelibrary.com]

All ECG time series, having been downsampled from 1200 Hz to 40 Hz, were input into the ridge extraction function, choosing a frequency range of 0.5–2 Hz in which to extract the IHR. This provided an output of time series of the variability of the heart rate over time of the same length as the original signal, one for each subject in each condition (i.e. NN, NH, etc.). For each time series, the mean, standard deviation and coefficient of variation values were calculated, and these values were then compared for significant differences between experimental conditions using the Kruskal–Wallis and rank‐sum tests, which compare the medians of the groups (see Fig. 3).

#### Respiration rates

The same procedure was followed as for the heart rates above, but the frequency range selected was 0.145–0.6 Hz (also see Fig. 3).

#### Wavelet transforms of respiration and instantaneous heart rates

All wavelet transforms were calculated using the wavelet transform option in the time frequency analysis function in MODA. The frequency range in which the transform was calculated was 0.005–2 Hz. The central frequency of the wavelet used was 1. Pre‐processing was set to ‘on’ (see MODA manual for further details). Respiration signals were downsampled from 1200 Hz to 40 Hz; IHR signals were already sampled at 40 Hz following ridge extraction. All respiration signals and all IHR signals for each subject in each condition were wavelet transformed, using the default wavelet function, which is a lognormal wavelet.

This provides, for each signal, a matrix of complex coefficients with information about both frequency and phase at each point in time. The amplitude coefficients were squared and averaged in time (mean), providing a time‐averaged wavelet power within the frequency range specified. These time averages were then averaged for all subjects for each condition, providing the graphs for mean wavelet power shown in Fig. 5*A* and *B*.

#### Phase coherence

The coefficients generated in the previous step are then used to calculate wavelet phase coherence using the phase coherence function in MODA. To calculate phase coherence between respiration and IHR, the IHR and respiration signals were input to the function for each subject in each condition. This provides the phase coherence in the same frequency range, 0.005–2 Hz, and also the phase difference at these frequencies. Again, the results were averaged in time for each subject and condition, and then averaged across subjects to provide the graphs shown in Fig. 5*C* and *D*. The same methods were used to calculate the phase coherence between the blood flow signals and the IHR and respiration shown in Fig. 8, although only the frequency range 0.03–0.5 Hz is shown.

The main differences in phase coherence between IHR and respiration were found in the frequency range 0.03–0.15 Hz, which is referred to as the modulation band. Single coherence values within this band were obtained by calculating the mean of the time‐averaged phase coherence for each subject in each condition within this frequency band. These single values were then used for comparison between conditions, as seen in Fig. 7, and for comparison with genetic results, as seen in Fig. 9.

#### Genetic results

Each subject underwent testing for the specific genes described above. The single, averaged phase coherence values obtained above from the modulation band were grouped according to whether the subject had a normal, heterozygous or homozygous (only in *NOTCH*) result. Significant differences in these groups were then investigated using the Kruskal–Wallis test.

### Statistical analysis

Iterative amplitude adjusted Fourier transform (IAAFT) surrogates were used in wavelet phase coherence calculations to assess significance. Wavelet phase coherence was considered significant if it was above the 95th percentile of 100 IAAFT surrogates (Schreiber & Schmitz, 2000). All phase coherence values presented have had this surrogate threshold subtracted. The statistical significance between groups was evaluated using non‐parametric tests, to avoid the assumption of normal distributions in the data. To compare between more than two groups the Kruskal–Wallis test was used and, if significant differences were found, they were further investigated using the Wilcoxon rank‐sum test. The null hypothesis for both tests is that the sample groups have the same median. All statistical tests were performed using MATLAB (The MathWorks Inc., Natick, MA, USA).

## Results

### Heart and respiration rates

Mean heart rates differed significantly between groups (*P* = 0.0000) (Fig. 3*A*). During sustained hypoxia (NH and HH) the heart rates were significantly higher than in the normoxic state (NN). However, they were not significantly higher than in normoxia during either acute normobaric (NH_a_) or acute hypobaric (HH_a_) hypoxia. Significant differences were also observed between the standard deviations of the IHR (*P* = 0.0005), shown in Fig. 3*C*. The variability of the heart rate was significantly higher during HH_a_ and HH than during normoxia, but there was no significant difference in variability between NH and NN. The coefficient of variation (CV) of the heart rate differed significantly between groups (*P* = 0.0453), and is shown in Fig. 3*E*. It is also known as relative standard deviation and is defined as the ratio of standard deviation of the heart rate to its mean. It was highest during HH_a_. It is obvious that the variability (standard deviation) of the heart rate is not linearly related to the actual heart rate.

Similarly, the instantaneous respiration rate was extracted by ridge extraction, and its mean and standard deviation over time were calculated. The group medians are shown in box plots in Fig. 3*B* and *D*. Mean respiration rates were significantly different between groups (*P* = 0.0000). The results show that the mean respiration rate significantly increased from normoxia during sustained hypoxic conditions, but not during acute hypoxia. Mean respiration rates did not differ significantly between prolonged NH, HH_a_ and prolonged HH. A significant difference was also observed in the standard deviation of the respiration rate (*P* = 0.0006). This was caused by a significant reduction in the standard deviation of respiration rate during NH_a_ only (Fig. 3*D*). The CV of the respiration rate (Fig. 3*F*) did not differ significantly between groups (*P* = 0.2931), indicating that the variability (standard deviation) of the respiration rate is almost linearly related to the actual respiration rate.

### Periodic breathing

**Figure 4:**
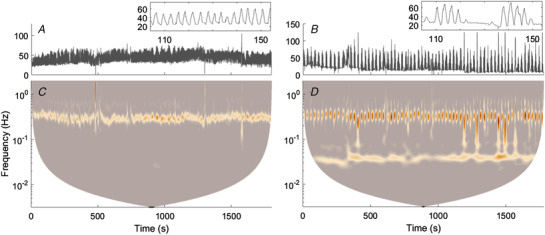
Continuous wavelet transforms of respiration signals during normoxia and normobaric hypoxia *A*, breathing signal from one subject during normoxia. *B*, breathing signal from the same subject during sustained normobaric hypoxia (NH). Insets in *A* and *B* show 50 s samples of each time series. *C*, wavelet transform of the respiration signal in *A*. *D*, wavelet transform of the respiration signal in *B*. [Color figure can be viewed at wileyonlinelibrary.com]

**Figure 5:**
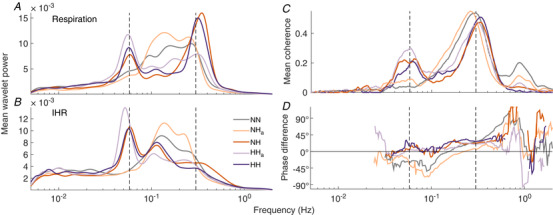
The time‐averaged wavelet powers for respiration and instantaneous heart rate after normalisation *A* and *B*, group means of the normalised time‐averaged wavelet transforms for respiration signals (*A*) and instantaneous heart rate (IHR) signals (*B*) for each condition. *B* describes the nature of heart rate variability. *C*, group means of the phase coherence between the respiration and instantaneous heart rate signal, with the critical surrogate threshold subtracted. *D*, group means of the time‐averaged phase difference for the coherence shown in *C* at each frequency, shown for significant coherence only. Dashed lines indicate 0.058 Hz, where an extra peak appears in both spectra during hypoxia, and 0.3 Hz, the approximate respiration frequency during hypoxia. NN, normobaric normoxia (*n* = 22); NH_a_, acute normobaric hypoxia (*n* = 22); NH, normobaric hypoxia (*n* = 18); HH_a_, acute hypobaric hypoxia (*n* = 16); HH, hypobaric hypoxia (*n* = 13). [Color figure can be viewed at wileyonlinelibrary.com]

A regular, low‐frequency oscillation at a frequency below that of breathing emerged intermittently in most subjects during sustained normobaric and hypobaric hypoxia, and was identified as arising from PB; an example respiration signal from one subject is shown in Fig. 4*B*, and its wavelet transform in Fig. 4*D*. The wavelet transform of the respiration signal during PB shows two distinct oscillations, one at the respiration frequency (∼0.35 Hz) and one at the modulation frequency (∼0.05 Hz). The frequency of modulation varied slightly between subjects and conditions but did not vary significantly (*P* = 0.799). Time‐averaged wavelet transforms of the respiration signals for all subjects in each state are compared in Fig. 5*A*. A low‐frequency peak is clearly visible around 0.058 Hz in the mean spectra of all hypoxic states except NH_a_, in addition to peaks at the respiration frequency (∼0.3 Hz in hypoxia). Note that the mean respiratory frequency is significantly higher during NH and HH conditions when compared to NN. Also at the respiration frequency, the group mean of the time‐averaged wavelet power of the respiration signal is higher in prolonged (NH, HH) than in acute (NH_a_, HH_a_) states.

### Heart rate variability

**Figure 6:**
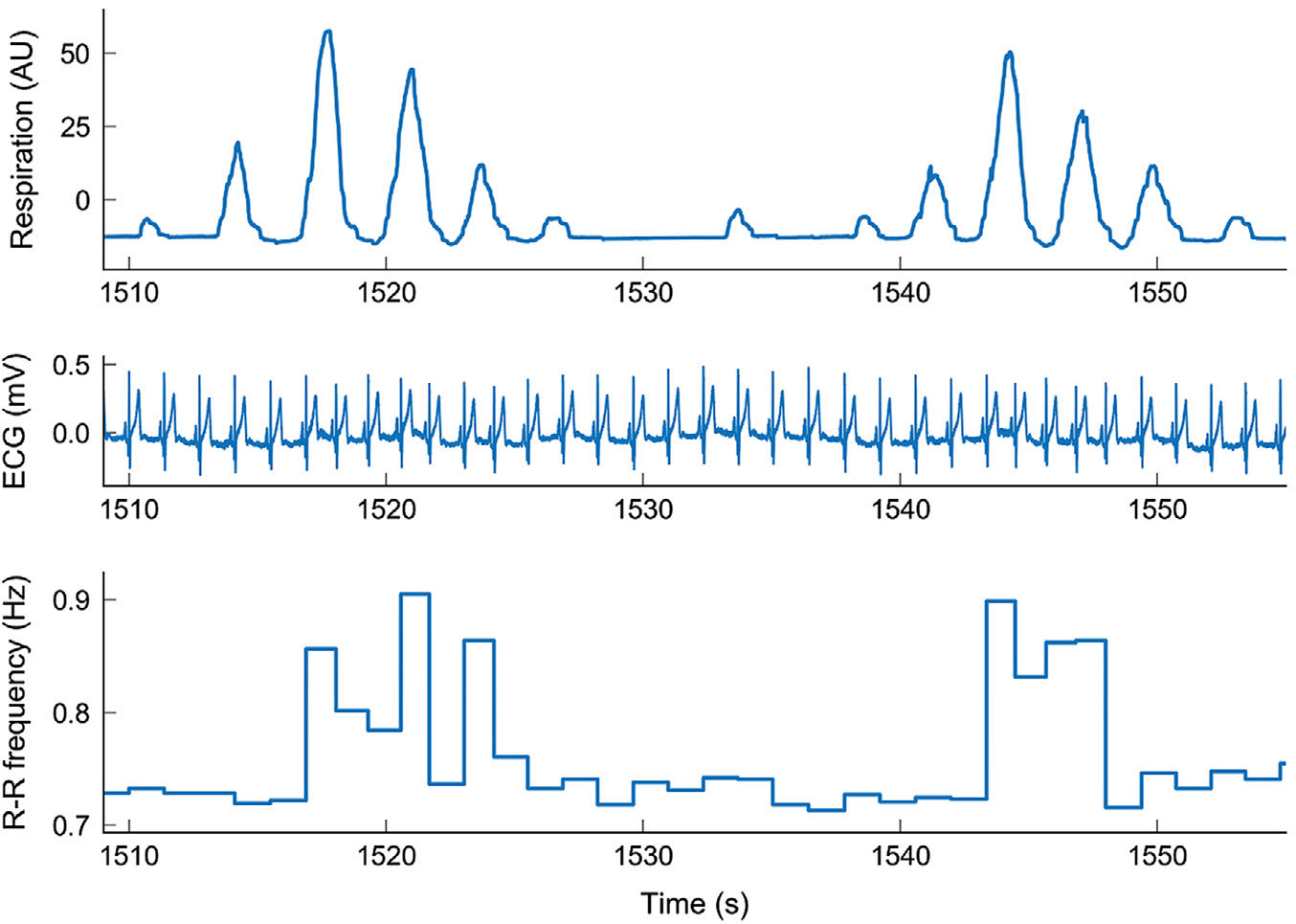
Respiration (top), ECG (middle) and heart rate (bottom) during NH‐induced PB in subject S15 For the sake of clarity, the heart rate was plotted directly from the ECG, defined as an inverse of the time between consecutive R–R peaks. The normal heart rate for this subject (during NN) is 0.76 Hz. It can be seen that the heart rate increases during the episodes of periodic breathing. [Color figure can be viewed at wileyonlinelibrary.com]

HRV was analysed in terms of the time‐averaged wavelet power spectrum of the IHR signal. The mean HRV for each group is compared in Fig. 5*B*. As also observed for respiration, a significant peak in the HRV around 0.05/0.06 Hz appears during all hypoxic states except acute normobaric hypoxia (NH_a_, orange line). This peak is more pronounced in HH_a_ and HH, which may explain the increased standard deviation of IHR observed in these groups. During hyperventilation the heart rate is usually higher than during apnoea, as illustrated in Fig. 6, similar to the results of Franklin *et al*. (1997) for Cheyne–Stokes respiration of patients with heart failure.

### Phase coherence for instantaneous heart rate and respiration

**Figure 7:**
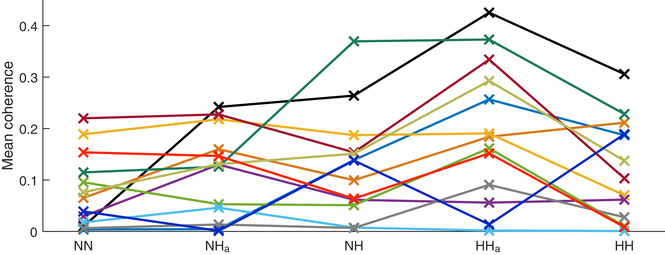
Phase coherence between respiration and instantaneous heart rate averaged over the periodic breathing modulation frequency band (0.03–0.15 Hz) for all states for the 13 subjects that attended all sessions NN, normobaric normoxia; NH_a_, acute normobaric hypoxia; NH, normobaric hypoxia; HH_a_, acute hypobaric hypoxia; HH, hypobaric hypoxia. The linear connections between points are presented purely to help identify visually the changes for individual subjects, each represented by a different colour. [Color figure can be viewed at wileyonlinelibrary.com]

To investigate the relationship between IHR and respiration, and whether or not the low‐frequency peaks observed in the spectra of the respiration and heart rate signals are related, wavelet phase coherence was calculated between the respiration signal and the IHR extracted from the ECG for each subject in each condition. This phase coherence will sometimes be referred to as cardiorespiratory (CR) coherence. Results are shown in Fig. 5*C*. A low‐frequency peak in the coherence can be observed clearly in the range 0.03–0.1 Hz in all states except normoxia. This indicates coherence at frequencies corresponding to that of the resulting modulation associated with PB. It suggests that the physiological mechanism involved in the modulation is simultaneously present in both signals. Although the general trend shows that IHR–respiration coherence is increased during hypoxia, this is not the case for all subjects. Results for the 13 individuals that attended all measurements are shown in Fig. 7.

The phase difference can also be used to determine which of two coherent oscillators is leading, and thus more likely to be the origin of the dynamics. The group means of the time‐averaged phase difference for each condition are shown in Fig. 5*D*. As would be expected due to RSA, the respiration signal leads within the respiratory frequency band (around 0.25 Hz under control conditions and up to 0.33 Hz in prolonged hypoxia), as demonstrated by the positive phase difference in all five conditions. In the modulation of respiration frequency band, the coherence is higher in all cases of hypoxia compared to normoxia. In this band the phase difference is slightly positive during hypoxia (with the exception of NH_a_ where the smallest power of PB was observed), indicating that the mechanism giving rise to the component in this band in both signals is driving the respiration slightly ahead of driving the heart rate.

### Blood flow

**Figure 8:**
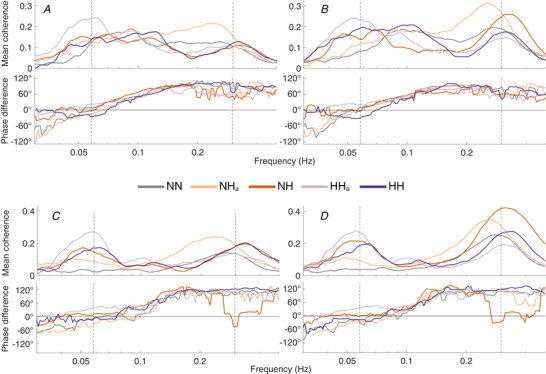
Group means of the phase coherence among instantaneous heart rate, respiration and LDF at the two wrists *A* and *B*, phase coherence between the instantaneous heart rate and the blood flow signal recorded using LDF in the left (*A*) and the right (*B*) wrists, in the frequency interval between 0.03 Hz and 0.5 Hz, all with the critical surrogate threshold subtracted. *C* and *D*, phase coherence between the respiration signal and the blood flow signal recorded using LDF in the left (*C*) and right (*D*) wrists, in the frequency interval between 0.03 Hz and 0.5 Hz, all with the critical surrogate threshold subtracted. For each of the four cases, the group mean of the time‐averaged phase difference is shown underneath the coherence plot. Dashed lines indicate 0.058 and 0.3 Hz, the approximate modulation and respiration frequencies, respectively. NN, normobaric normoxia (*n* = 22); NH_a_, acute normobaric hypoxia (*n* = 22); NH, normobaric hypoxia (*n* = 18); HH_a_, acute hypobaric hypoxia (*n* = 16); HH, hypobaric hypoxia (*n* = 13). [Color figure can be viewed at wileyonlinelibrary.com]

Low‐frequency oscillations were also observed in the time‐averaged wavelet transforms of skin blood flow recordings. To investigate their origins, wavelet phase coherence was calculated between the LDF signals and both the IHR and the respiration signals. Significant coherence was found in all comparisons, as shown in Fig. 8, together with the corresponding time‐averaged phase differences.

Coherence between the respiration and blood flow signals (Fig. 8*C* and *D*) was found at both the modulation frequency and the respiration frequency. Phase coherence at the modulation frequency was much smaller in the normoxic case, suggesting that this coherence may be due to the effects of the PB mechanism on the respiration and blood flow. In contrast, phase coherence between the IHR and blood flow (Fig. 8*A* and *B*) at the modulation frequency was high even during normoxia, suggesting that an oscillation at the PB modulation frequency may be present even during normoxia, albeit much weaker. The phase differences in Fig. 8 also yield the following important insights. In all four comparisons, there is a clear difference between the phase shifts at the respiration frequency and at the modulation frequency, with the blood flow clearly lagging behind in phase at the respiration frequency, except in the comparisons between respiration and blood flows in the case of prolonged normobaric hypoxia. This suggests that the periodic breathing has a different physiological mechanism from that of the normal respiratory cycle.

### Associations between genetic factors and periodic breathing

**Figure 9:**
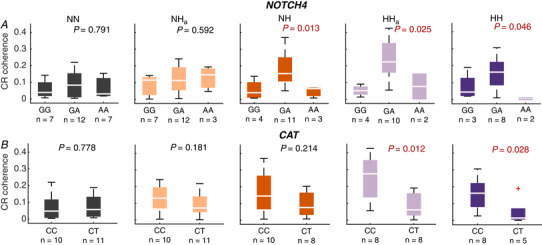
Boxplots of cardiorespiratory (CR) coherence in normoxia and hypoxia, grouped by genetic results for *NOTCH4* and *CAT* No significant differences were found during normoxia (NN) or acute normobaric hypoxia (NH_a_). During normobaric hypoxia (NH), the group of subjects with polymorphism in *NOTCH4* showed significantly higher CR coherence. In contrast, subjects with polymorphism in *CAT* showed lower CR coherence during HH and HH_a_. *P*‐values are results of the Kruskal–Wallis test. Significance is considered as *P* < 0.05, shown in red. AA, homozygous for polymorphism; GA, heterozygous; GG, normal; CC, normal; CT, heterozygous. [Color figure can be viewed at wileyonlinelibrary.com]

We have shown an increase in coherence between the IHR and respiration during hypoxia within the frequency band related to periodic breathing. The genetic information collected from each participant allows us to investigate whether any of the genes considered may be related to the hypoxic response. Mean CR coherence over the modulation band (0.03–0.15 Hz) was calculated for each subject in each of the five states, and divided into groups depending on the results for specific genes. No significant differences (in all cases *P* > 0.5) were found for the *SOD2*, *BDNF* and *GPX1* polymorphisms, suggesting that they are not related to cardiorespiratory phase coherence in the modulation frequency band. Results for the *NOTCH4* and *CAT* polymorphisms are shown in Fig. 9. Here, a significant difference was found for *NOTCH4* polymorphisms during hypoxia, with carriers of *NOTCH4* having significantly higher CR coherence, and thus more periodic breathing, in all hypoxic conditions except NH_a_. In contrast, significantly lower CR coherence was found during hypoxia in carriers of *CAT*. Interestingly, the significance of this difference was only observed during hypobaric hypoxia, both acute and prolonged, with no significant differences being found during normoxia or normobaric hypoxia.

The genetic results were also compared to the altitudes of the permanent places of residence of each subject in Table 2. Although it is difficult to quantify correlations, there appears to be a tendency for the normal results for *NOTCH4* (GG) to occur in those living at lower altitudes. There appear to be no trends with altitude in the *CAT* results.

**Table 2:** Table of altitudes of permanent residence for each subject, and the results of the genetic tests for *NOTCH4* and *CAT*

Subject	Altitude (m)	*NOTCH4*	*CAT*
17	199	GG	CT
18	199	GG	CT
20	235	AA	CT
15	260	GA	CC
1	262	GA	CC
11	295	GA	CC
12	295	GG	CC
16	295	GA	CT
21	295	GA	CT
22	295	GA	CT
4	295	GG	CT
5	295	GG	CT
6	295	GG	TT
7	300	GA	CT
13	380	GA	CC
14	380	GA	CC
8	380	GG	CC
9	380	GA	CT
2	466	GA	CC
3	492	GA	CC
10	531	AA	CT
19	683	AA	CC

AA, homozygous; CC, normal; CT, heterozygous; GA, heterozygous; GG, normal; TT, homozygous.

## Discussion

The aim of this work was to examine the effect of hypoxia on cardiovascular dynamics, including phase coherence and phase shifts between oscillations of the IHR detected from the ECG, of the respiration as detected from the respiratory effort, and the oscillations in the blood flow signal recorded by LDF. We also asked whether the cardiorespiratory coherence in the frequency interval of periodic breathing is related to polymorphism in specific genes. The four principal findings were: (1) normobaric and hypobaric hypoxia change heart and respiration rates, and their variabilities, (2) there is increased cardiorespiratory coherence during the episodes of periodic breathing, (3) the phase shifts between the IHR and respiration, on one side, and microvascular blood flow, on the other, are much smaller (almost zero) at the PB frequency, compared to the shifts at the respiration frequency, and (4) during hypoxia the cardiorespiratory coherence in the PB frequency interval is correlated proportionally to *NOTCH4* and is inversely proportional to the *CAT* polymorphism. Below we discuss the effects of hypoxia on various parameters related to cardiovascular dynamics.

### Heart and respiration rates and their variability

We have found significant differences in heart rates and respiration rates in subjects exposed to hypoxia, both normobaric and hypobaric. The hypoxia‐induced increase in resting heart rate is a known phenomenon, and seems to result from a decrease in vagal outflow and an increase in relative sympathetic activity (Cornolo *et al*. 2004; Botek *et al*. 2015). Significant differences were also found in the standard deviations of heart rates when comparing normoxia with both acute and prolonged hypobaric hypoxia, but not during normobaric hypoxia. The coefficient of variation indicated that the changes in heart rate variability (as quantified by their standard deviation) are not proportional to the changes of mean heart rate. Standard deviations of respiratory rates varied significantly from normobaric normoxia in all states but were almost proportional to the mean respiratory rates. These results demonstrate significant changes in the variation of heart and respiratory rates during hypoxia, and further investigations demonstrated that these are due, at least in part, to the presence of periodic breathing.

The identification of PB from time series is not a trivial problem, and usually requires the input of a specialist on a case by case basis. Similarly to Garde *et al*. (2015), we used the coherence between the respiration and the heart rate in the modulation frequency band to quantify PB during hypoxia. Because the heart rate is significantly influenced by the respiration via RSA, the distinctive periodic breathing pattern observed during hypoxia is likely to have an effect on the heart rate.

The direct effects of PB on the heart rate and blood pressure have been demonstrated using paced periodic breathing (Lorenzi‐Filho *et al*. 1999, Francis *et al*. 2000), demonstrating that alterations in heart rate are a direct effect of respiratory modulation, and can occur without the need for hypoxia. This highlights the importance of taking the breathing dynamics into account when investigating heart rate variability, even during normoxic conditions and, moreover, demonstrates that paced respiration directly alters the natural dynamics of the cardiorespiratory system, leading to a forced state.

It remains unclear whether the physiological responses to hypobaric (HH) and normobaric (NH) conditions are comparable (Millet *et al*. 2012). While a recent review (Coppel *et al*. 2015) suggested that the majority of cardiovascular variable responses were similar between HH and NH, there is clear evidence that hypobaria *per se* can independently influence certain aspects of ventilatory (Faiss *et al*. 2013) and cardiac dynamics (Netzer *et al*. 2017) as well as redox balance (Ribon *et al*. 2016) responses. Indeed, Heinzer *et al*. (2016) found that HH has a greater effect on nocturnal PB than does NH. The results obtained in our study show that the heart rate is significantly increased during both normobaric and hypobaric sustained hypoxic states when compared to the normoxic state. However, heart rates do not differ significantly between normobaric and hypobaric hypoxia, suggesting that this difference is most likely due to the limited oxygen availability rather than the atmospheric pressure itself. In contrast, the standard deviation of the heart rate is only significantly different during hypobaric hypoxia. This suggests that the differences in heart rate variability cannot be explained by oxygen partial pressure differences alone, and may be attributable to the effects of low barometric pressure on the physiology of the subjects.

### Phase coherence

Wavelet analysis was used to further investigate cardiorespiratory dynamics under different conditions. Both normobaric and hypobaric hypoxia induced PB, and we investigated the effects of this on the heart rate and the peripheral blood flow using wavelet phase coherence. Our results show that during PB, the heart rate is modulated not only at the respiration frequency, but also at the PB modulation frequency (see Fig. 5). This confirms previous findings by Garde *et al*. (2015) using time–frequency analysis to automatise the identification of PB.

We also demonstrated significant wavelet phase coherence during hypoxic conditions, at the modulation frequency between respiration and blood flow recorded at each wrist, showing that PB dynamics propagates throughout the cardiovascular system. It was previously shown that forehead skin blood flow in newborns oscillates with the rhythmicity of the breathing amplitude variation during PB (Jahnukainen *et al*. 1995). Our results also confirm the observations of Ovadia‐Blechman *et al*. (2017) using cross‐correlation analysis, which were attributed by the authors to a direct induction of vasomotion by slow respiration.

The increased CR coherence during periodic breathing could be an efficient adaptation to hypoxia. Possibly, increased CR coherence improves the efficiency of gas exchange. This finding could help in modelling the cardiorespiratory system, as the existing work (Ben‐Tal *et al*. 2012, 2014, 2019; Ben‐Tal & Tawhai, 2013; Molkov *et al*. 2014) assumed periodic breathing was maladaptive rather than beneficial.

Significant phase coherence was observed between the IHR and blood flow signals under all conditions. Using instantaneous phases, we were able to investigate phase differences and, for both respiratory and periodic breathing, the modulation phases showed a clear pattern relative to the blood flow recorded by LDF. Moreover, the pattern repeats for blood flow recorded on the left and the right arm. In all four comparisons, there was a clear difference between the phase shifts at the respiration frequency and those at the modulation frequency. While the blood flow is lagging behind at the respiratory frequency, there is minimal lag at the modulation frequency, indicating that the mechanisms underlying periodic breathing may operate at the microvascular level. This is further supported by the increased coherence at the PB modulation frequency observed in subjects with polymorphism in *NOTCH4*, which is involved in endothelial control of vascular modelling (Wu *et al*. 2005). Microvascular involvement may connect the observed dynamics indirectly to cellular respiration. While the exact mechanism remains to be elucidated, the results indicate that periodic breathing is of different physiological origin from the normal respiratory cycle.

In addition to the heart beat and breathing, several other lower‐frequency oscillatory processes are involved in the regulation of the cardiovascular system. Early studies of blood pressure regulation have identified ‘Mayer waves’, or ‘Traube–Hering–Mayer waves’ with a frequency of around 0.1 Hz in humans. They are associated with mechanisms of arterial blood pressure regulation with involvement of baroreceptors and chemoreceptors (Cherniack *et al*. 1969; Julien, 2006; Morris *et al*. 2010; Elstad *et al*. 2018). In the present study we based the analyses on the simultaneously recorded breathing, ECG and microvascular blood flow (also known as blood perfusion) measured by LDF. Most studies to date have investigated LDF leading to the identification of oscillations of myogenic, neurogenic and endothelial origin (Stefanovska, 2007). Blood vessels that contain vascular smooth muscle cells are able to constrict and dilate independently (known as vasomotion) in order to control blood flow locally. This myogenic activity manifests in the frequency interval 0.052–0.145 Hz. Vasomotion may also be initiated by local vessel sympathetic innervation, known as neurogenic activity (0.021–0.052 Hz (Söderström *et al*. 2003). The slowest oscillations in blood flow (0.005–0.021 Hz) have been shown to result from endothelial activity (Kvandal *et al*. 2006). These frequency intervals for local regulatory activity were considered by Paparde *et al*. (2015) in their investigation of the effects of hypoxia on cutaneous blood flow dynamics. Vascular‐related mechanisms of regulation are potential candidates for providing the underlying mechanisms of periodic breathing. The frequency of modulation during periodic breathing is, as shown in, for example, Fig. 8, around 0.058 Hz, most probably originating from the chemoreceptor‐related sympathetic activity. In the pattern of periodic breathing, Cheyne–Stokes respiration, associated with central sleep apnoea in patients with heart failure, the modulation frequency is lower, between 0.01 Hz and 0.03 Hz, and most probably has its origin in NO‐related endothelial activity – which is closely linked to cell energy metabolism. Alternatively, the Cheyne–Stokes respiration might have a similar origin as periodic breathing in infants, but be slower because of the much slower metabolism in subjects with heart failure. Further investigations are needed to elucidate which one of them is the main origin of the periodic modulation of breathing, and which of the others is/are involved indirectly via coupling mechanisms.

### Genetic correlates

Using cardiorespiratory phase coherence as a marker of PB, we have demonstrated a clear relationship between PB incidence during hypoxia and polymorphisms in *NOTCH4* and *CAT*, with the former being associated with a larger effect of PB and the latter associated with a smaller effect of PB. The NOTCH signalling pathway has previously been shown to be positively modulated by hypoxia (Hiyama *et al*. 2011). We also identified a potential link between polymorphism in *NOTCH4* and the altitude at which the subject resides, though this needs to be confirmed in a larger cohort.

### Methodological considerations

The data presented here were recorded from individuals who were all born pre‐term. Whilst this is not usually a factor that is considered during recruitment of healthy control participants for physiological research studies, it should be noted that pre‐term individuals that were exposed to intermittent hypoxia have an enhanced ventilatory response to hypoxia (Nock *et al*. 2004), and a more recent study showed a blunted hypoxic ventilatory response in the same group of participants presented in this paper (Debevec *et al*. 2019). Thus, it cannot be ruled out that the responses to hypoxia presented here are influenced by these effects.

Analyses of the relationship between PB coherence and the results of genetic testing were performed blind to experimental conditions.

### Significance and implications

High altitude physiology has been investigated for decades and has recently gained much attention (Davies *et al*. 2018; Ruggiero *et al*. 2018; Hoiland *et al*. 2019; Julian & Moore, 2019; Simpson *et al*. 2019). Clear physiological differences have been demonstrated comparing the physiological parameters of highlanders, from Himalaya (Ruggiero *et al*. 2018; Hoiland *et al*. 2019; Simpson *et al*. 2019) or Andes (Julian & Moore, 2019), with those of lowlanders, including faster recovery of peripheral muscle fatigue in Sherpas (Ruggiero *et al*. 2018), bringing phenotypical evidence for evolutionary adaptation in the control of cerebral blood flow and oxygen delivery at high altitude (Hoiland *et al*. 2019). A recent review suggested a need for further exploration of the interaction among genetic, epigenetic and environmental factors in shaping patterns of adaptation to high altitude. Such a study promises to improve the understanding of the mechanisms underlying human adaptive potential and to clarify its implications for human health. By identifying the involvement of *NOTCH4* and *CAT* we now provide evidence of specific gene polymorphisms related to the cardiorespiratory response to hypoxia. As *NOTCH4* is related to the functioning of the vascular endothelium (Wu *et al*. 2005), our study supports the recent observation by Simpson *et al*. (2019) that mechanisms other than peripheral chemoreflex activation contribute to vascular sympathetic resetting at high altitude. Furthermore, our study corroborates the findings by Davies *et al*. (2018) that differences in local microvasculature vasomotor and neurovascular control account for the ability of Sherpas to adapt to high‐altitude hypobaric hypoxia by sustaining local perfusion and tissue oxygenation. The results of our study extend these recent findings by postulating that even smaller differences in altitude may lead to different genotypes and hence to different responses to hypoxia.

Chronic periodic breathing is generally seen as an unfavourable state, being associated with increased mortality in chronic heart failure (Hanly & Zuberi‐Khokhar, 1996; Lanfranchi *et al*. 1999) and poor prognosis in patients with Cheyne–Stokes respiration compared to those without (Naughton, 1998), but it may have some advantages in healthy people at high altitudes (Küpper *et al*. 2008). Hypoxia, as well as occurring during acute ascents to high‐altitudes, is a significant problem at sea level, being a contributory factor in many pathological states including cancer (Eales *et al*. 2016), strokes (Hermann *et al*. 2007) and heart infarctions (Michiels, 2004). The similarities between hypoxia‐induced PB at altitude and the breathing characteristics observed in certain pathological states provides an opportunity to further our understanding of the physiological processes involved in chronic hypoxic states that occur even when oxygen is abundant. Considering living systems as collections of interacting oscillators whose dynamics are governed by multiple underlying open systems enables the observation of functional changes over time, and investigation into how they are altered in health and disease.

The term ‘periodic breathing’ is usually defined as clusters of breaths separated by intervals of apnoea (no breathing) or near‐apnoea, as opposed to normal breathing that is usually regular. However, in mathematics the term periodic function means a function returning to the same value at regular intervals. So, the term periodic breathing is misleading, because normal breathing is also periodic. As the intervals of regular breathing alternate with intervals of non‐breathing, or apnoea (or near‐apnoea), ‘periodic breathing’ can be seen as amplitude‐modulated regular breathing. Therefore, we propose a slightly more descriptive term ‘periodically modulated breathing’.

The polymorphism correlations with phase coherence between IHR and respiration that are shown in this study may be used to better understand a range of questions in cardiorespiratory physiology – from mechanisms of pathological breathing (Dempsey *et al*. 2010), to the physiology and enhanced performance of breath holding in elite apnoeists (Bain *et al*. 2018). This novel approach brings together genetics, vascular physiology and neural control of cardiorespiratory function, and has great potential to help advance physiology and pathophysiology quite generally.

### Conclusion

Quantification of periodic breathing during hypoxia using wavelet phase coherence provides a useful model for the study of low‐frequency respiratory modulation and its effects on the whole cardiovascular system. Using this approach, we have demonstrated clear associations between polymorphisms in *NOTCH4* and *CAT* and PB incidence. This provides specific evidence for a genetic basis for the wide variability in the hypoxic response and, crucially, identifies the specific genes involved. This study illustrates how one can uncover the link between structure at the genetic level and macroscopic mechanisms behind physiological phenomena.

## Additional information

### Competing interests

The authors declare no conflicts of interest.

### Author contributions

T.D., G.P.M., M.M., D.O., V.D. and A.S. contributed to conception of the study and design of the experiments. G.L., T.D., G.P.M., M.P., S.W., M.M., K.G., D.O., V.D. and A.S. contributed to the acquisition and analysis of data. G.L. and A.S. selected methods for analyses of oscillatory dynamics and G.L. performed the analyses. G.L., T.D., G.P.M., K.G., D.O., V.D. and A.S. contributed to the interpretation of data. G.L. drafted the manuscript and T.D., G.P.M., K.G., D.O., V.D. and A.S. edited and critically revised the manuscript. All authors have read and approved the final version of this manuscript and agree to be accountable for all aspects of the work in ensuring that questions related to the accuracy or integrity of any part of the work are appropriately investigated and resolved. All persons designated as authors qualify for authorship, and all those who qualify for authorship are listed.

### Funding

The work was supported by the Slovene Research Agency (Grant No. J3‐7536 and Program No. P20232), the Engineering and Physical Sciences Research Council (UK) (Grant Number EP/M006298/1) and the Innovative Training Network COSMOS program (funded by the EU Horizon 2020 research and innovation program under the Marie Sklodowska‐Curie Grant Agreement No. 642563).

**Translational perspective**

We tested the hypothesis that polymorphisms in selective antioxidative and neurodevelopmental genes are related to the mechanisms underlying the well‐known onset of periodic breathing during hypoxia, which are not yet fully understood. By extracting features of phase coherences between oscillations in the heart rate, breathing and blood perfusion of tissues during periods of normoxia and hypoxia, we confirmed the existence of correlations with phase coherence between IHR and respiration and polymorphism in *NOTCH4* and *CAT* during hypoxia‐induced periodic breathing. In this way we found a specific link between structure (genetics) and function (phase coherence in cardiorespiratory oscillations). Our methodology may be used to better understand a range of questions in cardiorespiratory physiology – from mechanisms of pathological breathing, such as sleep apnoea and Cheyne–Stokes breathing; to elucidating the link between genetics and function that results in differences in cardiorespiratory performance in highlanders and lowlanders; to establishing the link between function and genetics in reaction to hypoxia; and to the physiology and enhanced performance of breath holding in elite apnoeists, such as free divers.  Furthermore, mapping genes to function may help clinicians to understand and better programme therapies for many cardiorespiratory and vascular diseases.

## Supporting information


**Statistical Summary Document**.
