# Supplementary material for: Relationship between cardiorespiratory phase coherence during hypoxia and genetic polymorphism in humans
Source: J Physiol. 2020 Feb 26;598(10):2001–19. doi: 10.1113/JP278829 (PMC7317918; doi:10.1113/JP278829)
Supplement: Supplementary file 1 — Statistical Summary Document. [file TJP-598-2001-s001.docx]

**Manuscript Title: Relationship between cardiorespiratory phase coherence during hypoxia and genetic polymorphism in humans**

**Authors:** Gemma Lancaster, Tadej Debevec, Gregoire P. Millet, Mathias Poussel, Sarah J. Willis, Minca Mramor, Katja Goricar, Damjan Osredkar, Vita Dolzan, Aneta Stefanovska

%

**Animal model used, if applicable: NA**

**Underlying hypothesis:** Phase coherences and phase shifts among oscillations in the heart rate, respiration and the microvascular flow in hypoxic conditions are statistically linked with some of the common functional genetic polymorphisms in selective antioxidative (SOD2, CAT and GPX) and neurodevelopmental (NOTCH4 and BDNF) genes.

**Definitions of ‘n’:**

Subjects were measured on 5 occasions (all male):

1. NN – controls (normobaric normoxia); n=22,
2. NHa – acute normobaric hypoxia (simulated altitude); n=22,
3. NH – prolonged normobaric hypoxia (simulated altitude); n=18,
4. HHa – acute hypobaric hypoxia (real/terrestrial altitude); n=16,
5. HH – prolonged hypobaric hypoxia (real/terrestrial altitude); n=13.

The same subjects were included on all 5 occasions, although some dropped out because they could not travel from Ljubljana, Slovenia either to Planica, Slovenia or to Aiguille du Midi, France, or felt unwell because of prolonged hypobaric hypoxia at Aiguille du Midi.

**Statistical summary table:**

| Experimental question number* | Finding/ conclusion | Experimental location/ variable  e.g. cortex vs cerebellum or genotype | Mean value  (or other summary statistic) | SD | n (value) | P** | | Units | Data comparisons  e.g. WT vs KO | Statistical test | Any other variable  e.g. subjects’ age or sex | Figure/table in which data are presented | Comments  e.g. observation |
| --- | --- | --- | --- | --- | --- | --- | --- | --- | --- | --- | --- | --- | --- |
| 1 Do HRs differ between the 5 conditions listed above? | yes | ECG | Group median values were used. | NA | 22,22,18,16 and 13 | p = 0.0000 | Hz | | 1:2:3:4:5 | Kruskal Wallis test | NA | Fig. 3a |  |
| 2 Do respiratory rates differ between the 5 conditions listed above? | yes | Belt around the thorax | Group median values were used. | NA | 22,22,18,16 and 13 | p = 0.0001 | Hz | | 1:2:3:4:5 | Kruskal Wallis test | NA | Fig 3b |  |
| 3 Do CVs of heart rate (ratio of standard deviation of the heart rate to its mean) differ between the 5 conditions listed above? | yes | Same as 1 | Same as 1 | NA | Same as 1 and 2 | p = 0.0453 | Non-dimensional | | 1:2:3:4:5 | Kruskal Wallis test |  | Fig 3e |  |
| 4 Do CVs of respiratory rates differ between the 5 conditions listed above? | no | Same as 2 | Same as 2 | NA | Same as 1-3 | p = 0.2931 | Non-dimensional | | 1:2:3:4:5 | Kruskal Wallis test | NA | Fig 3f |  |
| 5 Is there a significant phase coherence between respiration and instantaneous heart rate averaged over the periodic breathing modulation band (0.03-0.15 Hz)? | Yes | ECG on the shoulders and lower rib and respiration measured with a belt around the thorax | Values between 0 and 1 | NA | 13 | Above 95^th^ percentile of 100 IAAFT surrogates for each subject in each condition | Non-dimensional | | Each of the 13 subjects in each condition | Wilcoxon rank-sum test | NA | Fig 7 |  |
| 6 Is the CR coherence related to NOTCH4 in each of the 5 conditions? | Yes, in NH, HHa and HH and no in NN and NHa | ECG and respiration, and blood samples | Values between 0 and 1 | NA | 22, 22, 18, 16 and 13 | NN p=0.8  NHa p=0.6  NH p=0.013  HHa p=0.025  HH p=0.46 | Non-dimensional | | GG:GA:AA | Kruskal Wallis test |  |  |  |
| 7 Is the CR coherence related to CAT in each of the 5 conditions? | Yes in HHa and HH and no in NN, NHa and NH | ECG and respiration, and blood samples | Values between 0 and 1 | NA | 22, 22, 18, 16 and 13 | NN p=0.8  NHa p=0.2  NH p=0.2  HHa p=0.012  HH p=0.03 | Non-dimensional | | CC:CT | Kruskal Wallis test |  |  | Same tests carried out for *SOD2* rs4880 and *BDNF* rs6265, but no differences obtained for any of the 5 conditions |
